# Multilayer fabrication of unobtrusive poly(dimethylsiloxane) nanobrush for tunable cell adhesion

**DOI:** 10.1038/s41598-018-37893-w

**Published:** 2019-02-12

**Authors:** Soo Sang Chae, Joo Hyun Jung, Won Jin Choi, Joung Kyu Park, Hong Koo Baik, Jongjin Jung, Hyuk Wan Ko

**Affiliations:** 1 Department of Materials Science and Engineering, Yonsei University, Seoul, 03722 Korea; 2 Department of Biochemistry, College of Life Science and Biotechnology, Yonsei University, Seoul, 03722 Korea; 3 Department of Chemistry, Hannam University, Daejeon, 34054 Korea; 4 College of Pharmacy, Dongguk University, Goyang, 10326 Korea; 5 Advanced Materials Division, Korea Research Institute of Chemical Technology (KRICT), Daejeon, 34114 Korea; 6 Present Address: Department of Nanostructured Materials, Leibniz Institute of Polymer Research Dresden, Dresden, 01069 Germany; 7 Present Address: Department of Materials Science and Engineering, University of Michigan, Ann Arbor, Michigan 48109 USA

## Abstract

Precise modulation of polymer brush in its thickness and grafting density can cause unexpected cell behaviors and regulated bioactivities. Herein, a nanoscale poly(dimethylsiloxane) (PDMS) brush was employed to use as a controllable material for cell adhesion. Facile fabrication of ultrathin monolayer PDMS nanobrush on an underlying substrate facilitated regaining cell adhesion through long-range cell attractive forces such as the van der Waals forces. We showed that cell adhesion is diminished by increasing the number of nanobrush layers, causing a gradual decrease of the effectiveness of the long-range force. The result demonstrates that ultrathin PDMS nanobrush can either promote or inhibit cell adhesion, which is required for various biomedical fields such as tissue-engineering, anti-fouling coating, and implantable biomaterials and sensors.

## Introduction

In the fields of cell culture and tissue engineering, tailoring the physicochemical properties of scaffold surfaces is very important for controlling cell attachment and morphogenesis in a wide range of applications such as in microfluidic systems, cell-based therapy, bone regeneration, and stem cell differentiation^1–3^. Surface modification with suitable chemical components is an efficient approach to modulating specific physicochemical properties such as stiffness^4^, wettability^5^, and morphology^6^. The polymer brush is particularly useful because it precisely introduces the designed chemical functionality to covalently grafted, high-density, and stable polymer chains while maintaining the innate properties of the scaffold substrates^7–10^. In addition, accurately controlled grafting density and chain length allow the transition of its micro-architectures and physical properties which cause unexpected cellular responses and bioactive features^11,12^.

Changing the thickness of the grafted polymer to a few nanometer scales can lead to unanticipated phenomena due to its physical interaction with the substrate materials^11^. Interesting physical eventuation was observed in other materials in nanoscale. For example, coating a substrate with a hydrophobic monolayer of graphene does not affect the intrinsic wetting behavior of the underlying substrate because of its extreme thinness called wetting transparency of graphene. This surprising phenomenon has been considered to originate from the long-range interaction of a substrate, called the van der Waals force, penetrating a graphene monolayer^13^.

For cell adhesion, there are diverse cellular interactions between a cell and the surface of a substrate such as the van der Waals force, electrostatic charge-charge interaction, hydrophobic interaction, and hydration effect^14,15^. Thus, the physicochemical properties of a scaffold substrate might influence the cell via various short-range (e.g., electrostatic interaction, hydration effect) and long-range interactions (e.g., van der Waals force). If we could make a very thin unfavorable polymer brush for cell adhesion to a few nanometers in thickness on a biocompatible substrate in order to interfere with these interactions between the cell and a scaffold substrate as exemplified by wetting transparency of graphene^13^, we might verify the effects of short- or long-range interaction on cell adhesion. Furthermore, if the nanometer thickness can be precisely controlled within such ranges, we can also determine how extensively each interaction could contribute to cell attachment. Since the hydrophobicity of poly(dimethylsiloxane) (PDMS) surface is not compatible for adherent cells despite those intensive use in microfluidic devices^16,17^, fabrication of nanometer-scale PDMS polymer brush might be useful for elucidating the importance of long-range cellular interaction with a biocompatible underlying substrate.

In this research, a facile fabrication method of a PDMS nanobrush by simply adjusting the number of the PDMS layer at the nanometer scale (approximately 4 nm thickness per layer) is presented. A monolayer of a PDMS nanobrush still exhibits high surface hydrophobicity comparable to that of a bulk PDMS, but it increases cell adhesion and proliferation in several mammalian cell lines. These results indicate that the nanoscale hydrophobic PDMS surface paradoxically provides favorable conditions for cell adhesion, which might be due to a long-range interaction between a cell and the underlying substrate. Moreover, we observed that the multilayer of the stacked PDMS nanobrush reduced cell adhesion. It supports the importance of a long-range interaction to work as a driving force to govern cell behaviors on ultrathin PDMS nanobrush.

## Results and Discussion

**Figure 1:**
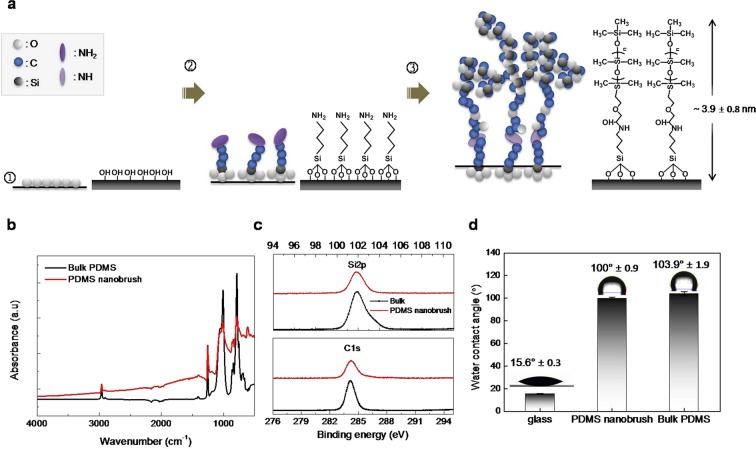
Fabrication scheme and material characteristics for the PDMS nanobrush layer on a glass substrate. (**a**) Schematic process and corresponding chemical structures. The PDMS nanobrush layer is fabricated on a glass substrate using an amine-epoxy reaction (①: Oxygen plasma, ②: 3-aminopropyl trimethoxysilane (APTES), ③: monoglycidyl ether-terminated PDMS (n ~ 6,000) at 80 °C for 4 hours). (**b**) Attenuated total reflectance (ATR) and infrared (IR) spectra of bulk PDMS (black) and PDMS nanobrush (red). (**c**) High-resolution X-ray photoelectron spectroscopy (XPS) Si2p (top) and C1s (bottom) narrow spectrum for bulk PDMS (black) and PDMS nanobrush (red). (**d**) Water contact angle for the glass substrate, PDMS nanobrush, and bulk PDMS. All measurements were replicated on at least three different samples (n_min_ = 3) and error bars denote standard deviations (SD) of the means.

The overall schematic process and material characteristics for the fabrication of the ultrathin PDMS nanobrush layer are presented in Fig. 1a^18^. Aminosilane was grafted onto the glass substrate by treating the surface with oxygen plasma and then immersing it in a 0.5 wt % aqueous solution of 3-aminopropyl triethoxysilane (APTES) for 10 min. Unreacted APTES on the surface was removed by agitation in deionized (DI) water. Then, the ultrathin PDMS nanobrush was generated via the epoxy-amine reaction by heating a droplet of PDMS precursor terminated with monoglycidyl ether at 80 °C for 4 h. The unreacted PDMS precursor was removed by washing three times with isopropyl alcohol (IPA), and the transparent and ultrathin PDMS nanobrush layer remained.

Spectroscopic ellipsometry results showed that the PDMS nanobrush layer was uniformly deposited with 3.9 nm thickness on the glass substrate (Fig. S1)^19,20^. Infrared (IR) spectroscopy (Fig. 1b) and X-ray photoelectron spectroscopy (XPS) did not detect any differences in the binding energy spectra of Si2p (top in Fig. 1c) and C1s (bottom in Fig. 1c) between the PDMS nanobrush and the bulk PDMS surfaces. IR spectroscopy and XPS indicated that the chemical composition of PDMS nanobrush was the same as that of the bulk PDMS, although the thickness of both PDMS substrates caused different baseline intensities.

Water wettability is the most crucial physicochemical property for cell adhesion and growth, that determines the cell fate on prepared matrices^5^. The PDMS nanobrush layer had high hydrophobicity, which was comparable to that of the bulk PDMS (Fig. 1d). The measured contact angles, which are good indicators of hydrophobicity, were 100.0° and 103.9° for the PDMS nanobrush layer and the bulk PDMS, respectively. Both displayed high hydrophobicity as compared to hydrophilic glass (15.6°). Scanning electron microscope (SEM) images showed a smooth surface without any noticeable features on the PDMS nanobrush layer and the bulk PDMS (Fig. S2).

**Figure 2:**
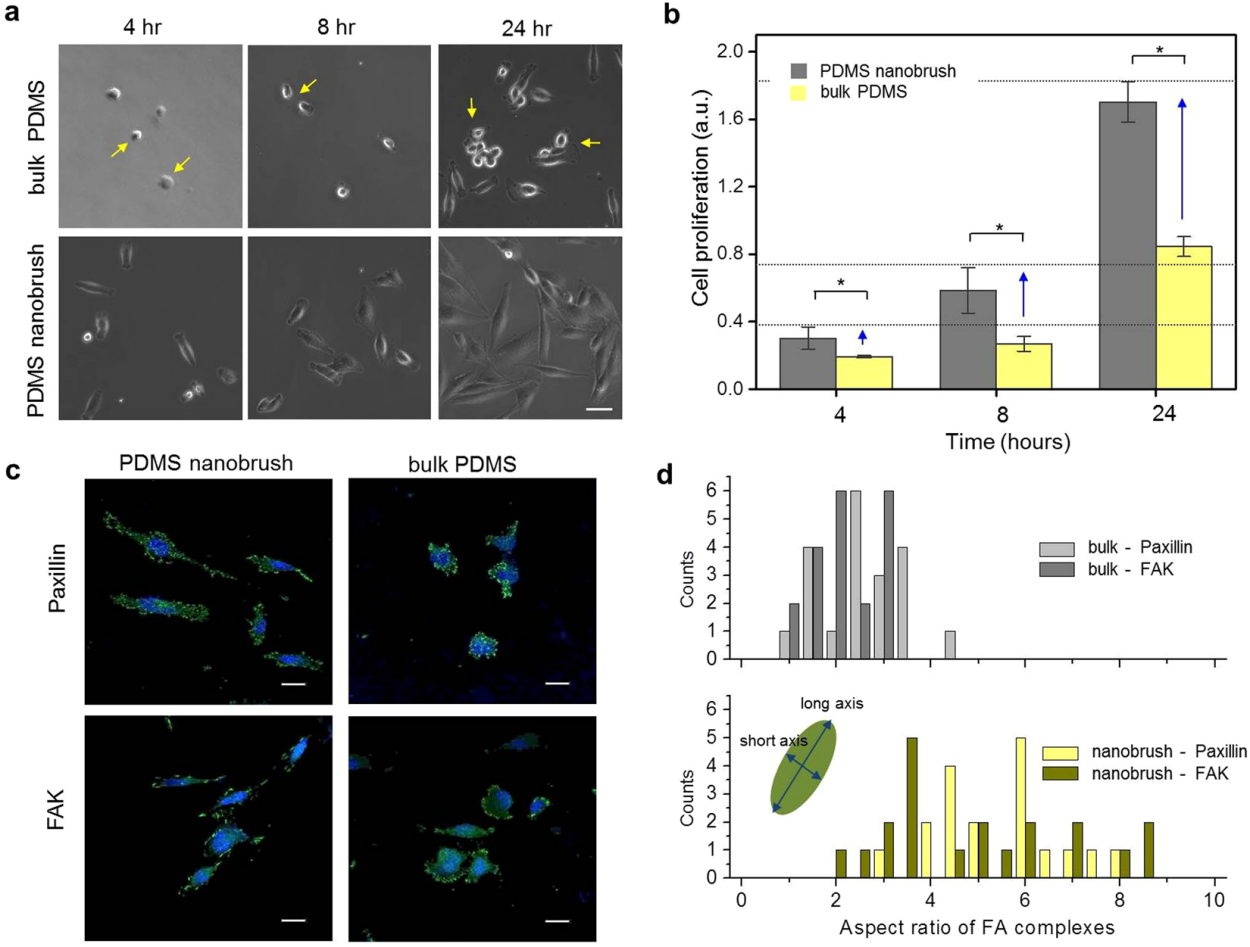
Hydrophobic PDMS nanobrush promotes SF295 glioma cell adhesion. (**a**) Photomicroscope images of cultured SF295 cells on either the PDMS nanobrush monolayer or bulk PDMS. Arrows represent the round shaped cells which are indicative of weak cell attachment to the substrate (scale bar = 40 μm). (**b**) Measurement of viable cells on indicated surfaces using MTS assay. The graph displays the means from triplicate independent experiments; error bars represent the standard errors of the mean, SEM (n = 3). Data were analyzed using one-way ANOVA with Tukey’s test (*p < 0.05). (**c**) Confocal immunofluorescence images of SF295 cells plated on the PDMS nanobrush and the bulk PDMS. Fixed cells were stained with antibodies against Paxillin (green) and FAK (green) to observe the formation of focal adhesion complexes. Nuclei were counterstained with DAPI (blue) (scale bar = 20 μm). (**d**) Quantitative measurement of the aspect ratio of FA complexes of cells grown on the bulk PDMS (top) or the PDMS nanobrush (bottom). 20 cells were used to measure FA parameters.

Next, we tested the cell behavior on the ultrathin PDMS nanobrush (nanoscale) and the bulk PDMS (millimeter scale) using a SF295 glioma cell line (Fig. 2). The two surfaces were equally hydrophobic with similar apparent wettability (greater than 100°, Fig. 1d); however, cell adhesion, spreading, and proliferation were significantly different on both surfaces. Hydrophobic surfaces do not generally provide a suitable condition for cell growth, and proper cell spreading and proliferation were suppressed on the hydrophobic surface of the bulk PDMS^21^. Interestingly, cell growth on the monolayer PDMS nanobrush was significantly higher than that on the bulk PDMS, but it was similar to that on the bare hydrophilic glass (Fig. S3a) although the fabrication of the monolayer PDMS nanobrush does not increase its roughness^11^ to the extent of offsetting the suppression of cell proliferation caused by its hydrophobicity (Fig. S4). To assess the stiffness/elasticity effect of the bulk PDMS toward the decrease of cell viability, surfaces of both PDMS substrates were coated with laminin, an extracellular matrix (ECM) protein, for an improved cell attachment in the initial seeding phase and a suitable culture condition^17^. Quantitative results of cell viability on laminin-coated PDMS substrates by MTS assay revealed that the stiffness/elasticity of the bulk PDMS slightly affects its suppressed cell growth even under extended period (72 hours) (Fig. S3b). It implies that cell adhesion and morphogenesis in the early stage, within 24 hours, determine the distinction of SF295 cell behaviors on both PDMS substrates such as cell proliferations.

Photomicroscope images of initial cell attachment and subsequent spreading of SF295 glioma cells were captured for 24 hours (h) to analyze cell behavior on the two surfaces (Fig. 2a). Cells on the monolayer PDMS nanobrush displayed robust adhesion to the surface, and proliferating cells formed normal cellular morphologies. By contrast, cells did not attach to the bulk PDMS surface, and proliferated cells displayed rounded cell morphologies. Different mixing ratios for the bulk PDMS may bring about a very minimal change in its characterization^17^. The MTS assay for cell growth and viability was performed for 4 to 24-h after cell seeding, and the results indicated that the PDMS nanobrush surface promoted greater cell proliferation than the bulk PDMS surface (Fig. 2b). At 4-h after seeding, there were no significant differences in the number of cells that adhered to the PDMS nanobrush and the bulk PDMS surfaces. At 24-h after seeding, the SF295 cell density on the PDMS nanobrush was approximately two times higher than that on the bulk PDMS.

We observed similar results with different types of cell lines such as HeLa (data not shown), NIH3T3, hMSC, and CAD (Cath.a-differentiated). The seeding and morphogenesis of these cell lines are analogous to that of SF295 cell line in these optical images (Fig. S5). On the other hand, cell viability assay of CAD seems to deviate from the tendency of SF295 and NIH3T3 on both PDMS substrates (Figs S3a and S6). Based on the microscopic images of CAD cells in Fig. S5, its unexpected proliferation rate on the bulk PDMS seems to arise from the abnormally aggregated cells anchored on its surface. All these data suggest that the ultrathin monolayer PDMS nanobrush improves cell attachment, morphogenesis, and survival compared with that of the unfavorable hydrophobic surface of the bulk PDMS.

Immunoflourescence staining was used to analyze the formation of cellular focal adhesion (FA) complexes on both surfaces using antibodies against Paxillin and focal adhesion kinase (FAK) and imaged by confocal microscopy (Fig. 2c and Fig. S7). Paxillin and FAK are major components in FA complexes that contribute to cell adhesion, migration, and proliferation^22^. Immunofluorescence images revealed remarkable differences in cell morphologies and FA complex formation between the PDMS nanobrush and the bulk PDMS surfaces. Quantitative comparisons indicated that the aspect ratio of the FA complexes was lower on the bulk PDMS, whereas elongated/ellipsoidal shapes of that on the monolayer PDMS nanobrush led to its high aspect ratio and enabled normal cell morphogenesis (Fig. 2d and Fig. S8). These results suggest that FA complex formation is suppressed in cells grown on the hydrophobic bulk PDMS surface, which results in abnormally rounded cell morphologies.

**Figure 3:**
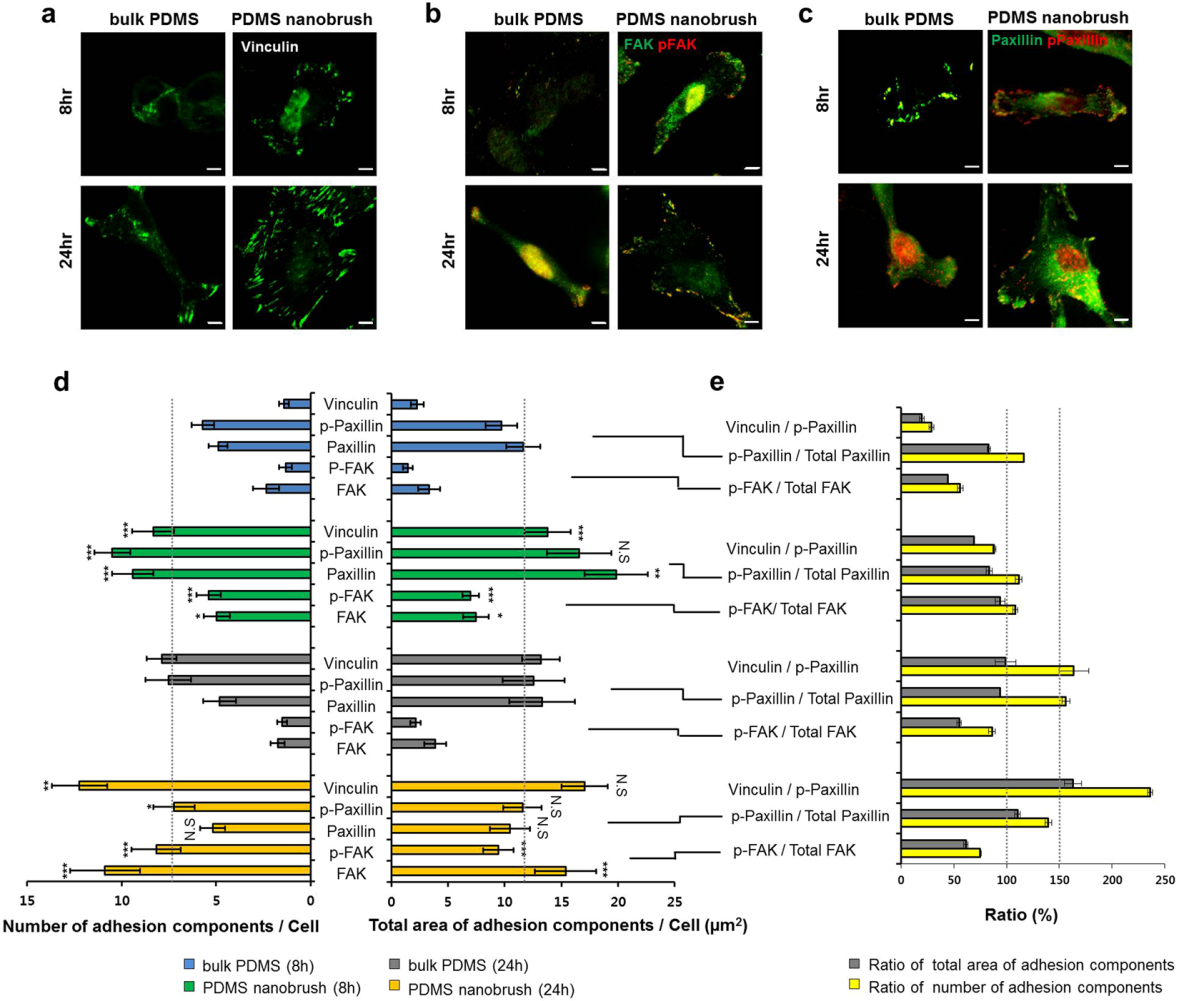
Immunofluorescence images of cell adhesion components and its quantitative analysis. Confocal immunofluorescence images of SF295 cells on the PDMS nanobrush and the bulk PDMS for 8-h and 24-h. Fixed cells were stained with antibodies against (**a**) Vinculin (green), (**b**) FAK (green) and phospho-FAK (red), and (**c**) Paxillin (green) and phospho-Paxillin (red) (scale bar = 5 μm). (**d**) Total number and the total area of adhesion components per cell were quantified via the processing of each image with ImageJ as previously described^28^. (**e**) Vinculin/p-Paxillin, p-Paxillin/total Paxillin, and p-FAK/total FAK were calculated for further analysis of cell adhesion state. 20 to 32 cells were used to measure cell adhesion parameters. The graph displays the means from triplicate independent experiments; error bars represent standard errors of the mean, SEM (n = 20 ~ 32). Data were analyzed using one-way ANOVA with Tukey’s test (*p < 0.05, **p < 0.01, ***p < 0.001, N.S: not significant). Statistical significances were evaluated for the following pair sets: the bulk PDMS vs the PDMS nanobrush (8-h) and the bulk PDMS vs. the PDMS nanobrush (24-h).

We further examined the cell adhesion state on both PDMS substrates at different times (8 and 24 hours after seeding) by staining FAK, phospho-FAK (p-FAK), Paxillin, phospo-Paxillin (p-Paxillin), and Vinculin with fluorescent antibodies (Fig. 3). Generally, cell adhesion starts from a weak adhesion stage (initial attachment) after seeding and enters into a strong adhesive stage by stable focal adhesion and fibrillar adhesion through intermediate adhesion (cell shape and spreading)^23^. Initial weak adhesion stage forms nascent adhesions which have dot-like shapes and transient lifetimes. On the other hand, adhesion maturation proceeds toward a strong adhesive state during a more extended period by forming stable focal adhesions and fibrillar adhesions with elongated shapes and longer lifetimes^24^. In the stages of cell adhesion before the maturation stage, adhesion components such as FAK and Paxillin are known to be highly phosphorylated, whereas focal adhesion and fibrillar adhesion in the maturation stage show low- level phosphorylation of FAK and Paxillin accompanying an abundant Vinculin^22,24,25^. Consistently, the changes in staining patterns of adhesion complexes follow previously stated in a time-dependent manner as shown in Fig. S9.

Furthermore, we examined molecular complexity of FA complexes of SF295 cells on PDMS substrates by immunofluorescently staining with various FA components (Fig. 3). We found that Vinculin signals are upregulated and localized to FA complexes on the PDMS nanobrush substrate cultured for 24-h. (Fig. 3a). The ratio of Vinculin to p-Paxillin which represents the extent of adhesion maturation process is also high for FA complexes on the PDMS nanobrush (24-h). SF295 cells on both the PDMS nanobrush (8-h) and the bulk PDMS (24-h) show a moderate amount of Vinculin and its ratio to p-Paxillin (Fig. 3f,e). These results indicate that a quite significant number of cells on the PDMS nanobrush (24-h) might undergo adhesion maturation stage.

Different from the expected results of Vinculin staining, other parameters such as downregulation of phosphorylation of Paxillin and FAK did not show significant differences for the evaluation of focal adhesion maturation stage. For example, the ratios of p-Paxillin to total Paxillin seem to be similar for all FA complexes on both PDMS substrates at different times although the quantification of Vinculin/p-Paxillin for all substrates is quite distinct (Fig. 3e). Given that the amount of p-FAK and FAK are highly suppressed for FA complexes jut on the bulk PDMS substrate, p-FAK/FAK could not be a good indicator for FA maturation due to its plausible dependency on other factors such as the properties of the substrate material.

Considering the ratio of the amount of Vinculin and its ratio to p-Paxillin and cell morphologies and cell proliferation gross rate, the PDMS nanobrush (24-h) is likely to be in the focal adhesion maturation stage under a relatively strong adhesive state despite the inconsistent p-FAK/total FAK and p-Paxillin/total Paxillin patterns. The bulk PDMS (8-h) clearly corresponds to the initial adhesion step under a weak adhesion state and other PDMS substrates at different times presumably initiate the maturation process.

**Figure 4:**
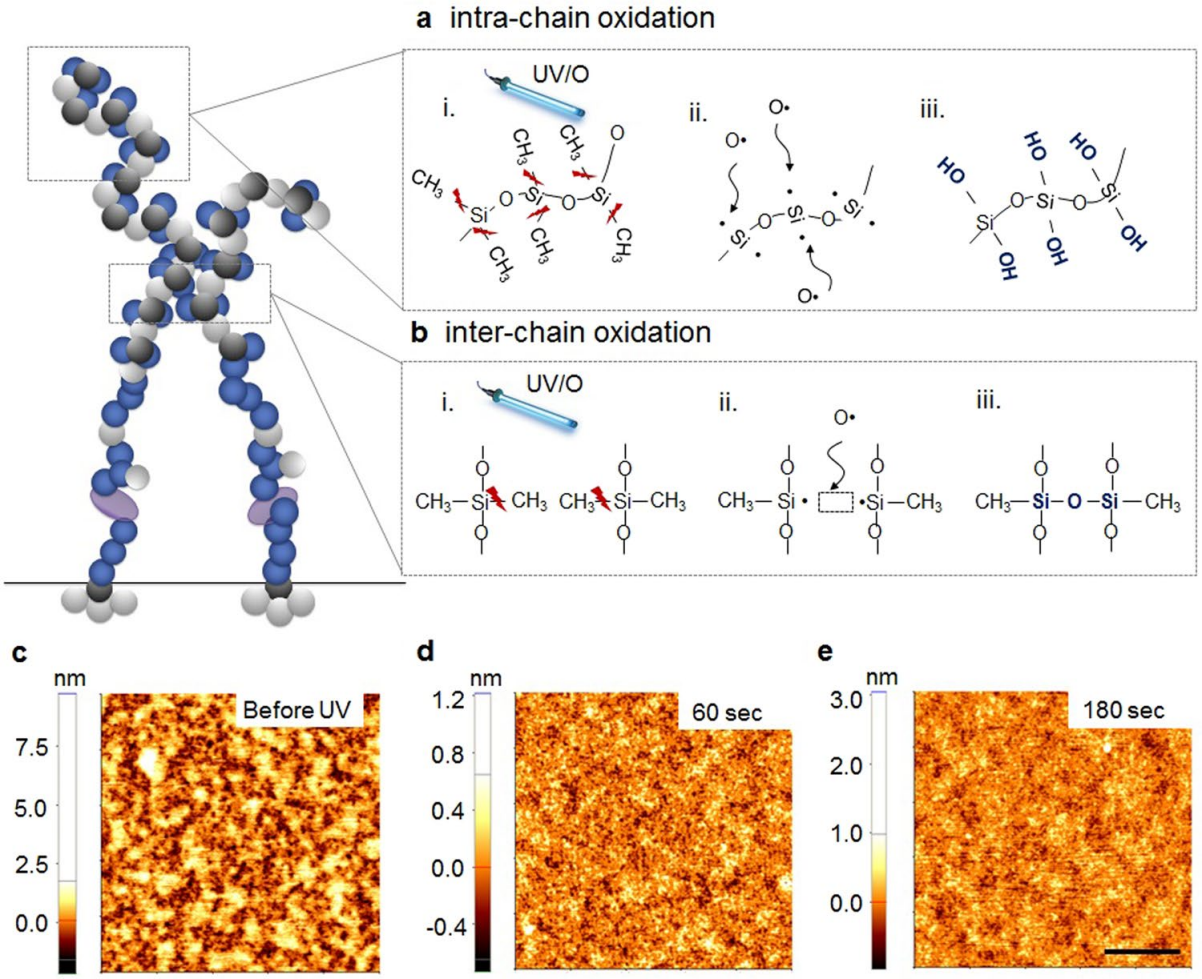
Proposed chain structures of mono- and bi-layer PDMS nanobrush. Photochemical oxidation of PDMS nanobrush for stacking multilayer *via* (**a**) intra-chain and (**b**) inter-chain reactions. (**c**–**e**) Atomic force microscopy (AFM) images of the PDMS nanobrush layer with increasing UV irradiation time. (**c**) As-deposited PDMS nanobrush. Upon UV exposure for (**d**) 60 sec and (**e**) 180 sec (scale bar = 0.5 μm).

Despite its indistinguishable hydrophobic tendency from the bulk PDMS, FA complexes relatively formed well on the ultrathin PDMS nanobrush. This phenomenon might attribute to the long-range interaction (e.g., van der Waals force) between cell adhesion-favorable underlying substrate and FA complexes. However, in case of the monolayer PDMS nanobrush, short-range interactions might influence cell adhesion by binding layer-penetrating cell adhesion molecules such as an integrin protein to the background. To differentiate the effect of each force (e.g., short-/long-range force), we have newly developed a fabrication method by stacking multiple PDMS nanobrush layers through partial photo-oxidation on UV exposure (Figs 4 and S10)^26^.

When UV photochemically oxidizes the PDMS nanobrush, firstly, Si-CH_3_ bonds in the backbone chain are scissored by a photo-energy of the UV light. This reaction is believed to occur entirely in the whole PDMS nanobrush. However, at the next stage, it might be considered that different responses arise in regions where the nanobrush chain is in contact with its neighboring nanobrush chain and in the area in which the chain is independent on an individual single chain. In case of the latter, the cleaved Si- bond reacted with a nearby oxygen radical and the generated Si-O• readily hydrated to stabilize its state (Si-O•), leading to the formation of Si-OH.

This reaction is called the ‘intra-chain’ photochemical oxidation in the PDMS nanobrush (Fig. 4a). On the other hand, when a PDMS chain is adjacent to another neighboring PDMS chain, two Si- bonds occur in the neighboring chain to form a Si-O-Si bridge, which share one oxygen radical because the neighboring chains of the nanobrush is close enough for the linking reaction. This reaction is called the ‘inter-chain’ photochemical oxidation in the PDMS nanobrush (Fig. 4b).

These photochemical oxidation reactions can be found through AFM image as a function of UV exposure time (Fig. 4c–e). Morphology is largely different after the photochemical oxidation of the PDMS nanobrush. With increased duration of UV light exposure, the pores (dark spots) decreased whereas hydroxyl group (–OH) areas (bright spots) became clear. It strongly implies that the inter-chain oxidation formed the interconnected PDMS chains that covered the defected areas. In addition, the XPS spectra of Si 2p in Fig. S11 show the quantitative formation of photo-oxidized SiO_x_ that increased as photo energy of UV was accumulated.

**Figure 5:**
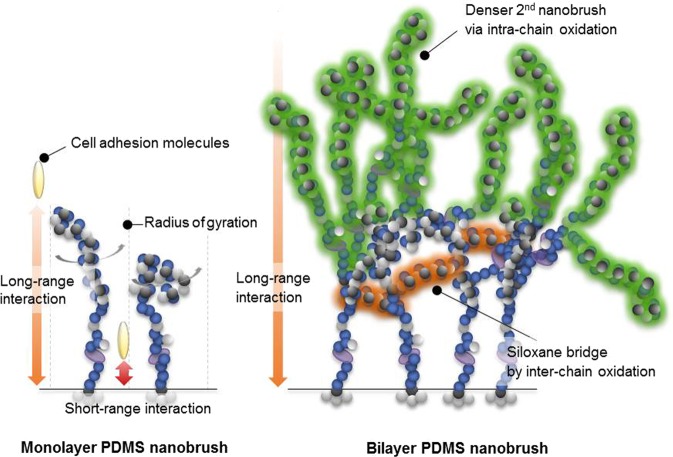
Proposed chain structures of mono- and bi-layer PDMS nanobrush. Different chain structures in the monolayer PDMS nanobrush (left) and the bilayer (or multilayer) PDMS nanobrush (right). The bilayer PDMS nanobrush has completely shielded the short-range interaction.

When the second PDMS nanobrush forms after the photochemical oxidation of the first PDMS nanobrush layer, a denser chain structure is formed which can entirely prevent cell adhesion molecules to penetrate to the underlying substrate *via* short-range interaction. This is based on the following two reasons. First, numerous hydroxyl groups (–OH) also formed along the main chain of the first PDMS nanobrush (*via* intra-chain photochemical oxidation) can act as a grafting site on the second PDMS nanobrush. Second, the siloxane bridge bonding via inter-chain photochemical oxidation builds a very stable and strong horizontal silica network, causing it tougher for cell adhesion molecules to penetrate (Fig. 5).

**Figure 6:**
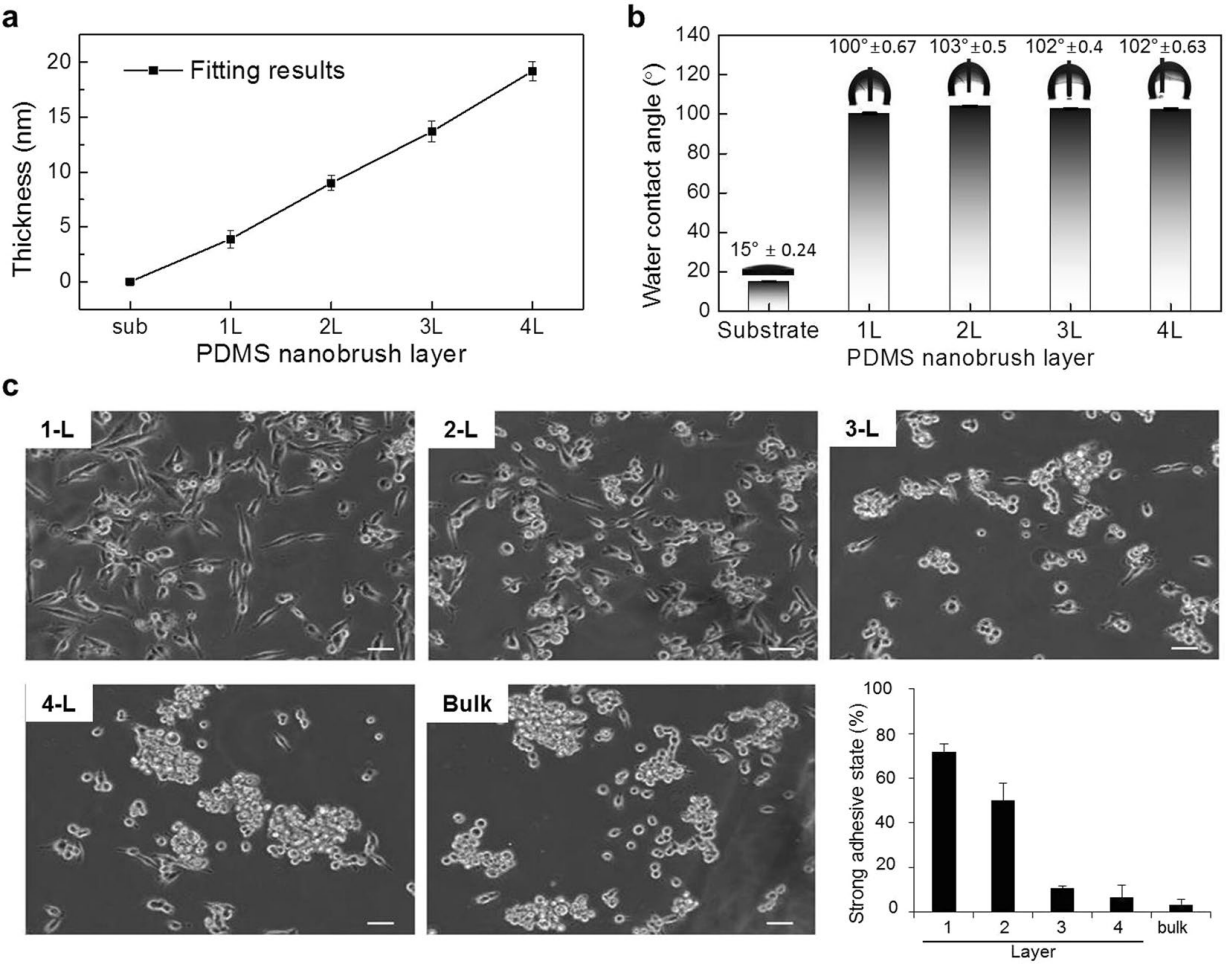
Control of cell adhesion using stacked layers of PDMS nanobrush. (**a**) Thicknesses of multiple PDMS nanobrush layers. (**b**) Water contact angles with increasing numbers of PDMS nanobrush layers. All the experiments were performed on at least three different samples and error bars represent the standard deviation (n_min_ = 3). (**c**) Phase-contrast images and quantification of adhered cells on varying layers of the PDMS nanobrush surfaces after 18 hours of cell seeding (scale bar = 100 μm). The percentage amount of strong adhesive states of cells in spreading shape was normalized to the total cell numbers (bottom right panel). To evaluate the ratio of strongly adhered cells, more than 100 cells for each substrate were analyzed to the total number of adhered cells. The histogram shows the means from three different image data; error bars represent the standard deviation, SD (n = 3).

Next, we examined the cell behaviors on the stacked multiple PDMS nanobrush layers, from a monolayer (3.9 nm) up to four layers (~20 nm) (Fig. 6a and Fig. S10). The same level of contact angles from a monolayer to four layers indicated that the terminal chemical functional group of all multilayers and monolayers are methyl groups (–CH_3_) (Fig. 6b). We used these multilayers of PDMS nanobrush to verify the long-range cellular interaction by comparing the extent of cell adhesion state. The process of cell adhesion to a surface material is subdivided into three stages: attachment, spreading, and formation of focal adhesions and stress fibers^23^. Cells undergo morphological changes to spread and a maturation process of FA complexes^24^ in strong adhesive states. Interestingly, the extent of cell adhesion was inversely correlated with the number of stacked PDMS nanobrush layers (Fig. 6c). Notably, the bilayered PDMS nanobrush enabled about half of the cells to remain in strong adhesive state even though it shielded off other possible short-range interactions *via* photo-oxidation reaction as described above.

Over bi-layer PDMS nanobrush, the relative percentages of strong adhesive cell state sharply decreased as the number of layers increased. When it exceeds four layers of the PDMS nanobrush, the percentage of strong adhesive cell states on the PDMS nanobrush was almost equal to that on the bulk PDMS. Thus, these results indicate that the long-range interaction such as the van der Waals force mainly contribute to the strong adhesive cell state in the mono-/bi-layer PDMS nanobrush, and those interactions are gradually suppressed with increased distance between the cell and the underlying substrate. In addition, the ratio of the strong adhesive state to the total number of attached cells appears to be strongly correlated to its proliferation (Fig. S12). As a result, the precisely controlled fabrication method of the multilayer nanobrush within the nanometer-scale revealed that the effective range of the long-range interaction is about 20 nm in thickness in case of SF295 glioma cell line. Because different types of cell lines show varying characteristics for cell adhesion and growth^17,27^, the spectrum of the effective long-range interaction can be altered within a limited distance. It resulted in different cellular responses according to cell types on the four layered PDMS nanobrush (around 20 nm in thickness) (Fig. S5). Cell dependency on the thickness of the nanobrush layers confirms that cell attachment, spreading and growth can be modulated by adjusting the thickness via long-range interaction regulation.

## Conclusion

This research showed the fabrication of multilayered ultrathin PDMS nanobrush and the dependence of cell behavior on the number of layers. With the mono/bilayered PDMS nanobrush, cells properly adhered and formed FA complexes on the substrate despite of their strong hydrophobic tendency comparable with that of the bulk PDMS which is well-known unfavorable substance for cell adhesion. With the increasing number of the PDMS nanobrush layers to tri-/tetra layers, cell adhesion significantly decreased. These results provide experimental evidence that a long-range interaction between a cell and the underlying substrate exists and it contributes to the dependence on the ultrathin multilayered PDMS monolayer. We believe that the multilayered PDMS nanobrush layer with tunable cell adhesion can represent a new class of ultrathin cell adhesion layer for synthetic implant surfaces that require precise nanoscale roughness in which conventional thick polymeric adhesion layer cannot be used.

## Experimental Details

### Fabrication of the Mono/Multilayer PDMS nanobrush and the bulk PDMS

The rectangular glass substrates were treated with O_2_ plasma (55 W) for 60 s at room temperature. Then, aminosilane was anchored on the glass by immersion in a 0.5 wt% aqueous solution of 3-aminopropyl trimethoxysilane (APTES) (Aldrich) for 30 min at room temperature. The unreacted APTES was washed away with distilled water. The ultrathin PDMS nanobrush was produced via the epoxy-amine reaction by dropping a sufficient volume of monoglycidyl ether-terminated poly(dimethylsiloxane) (PDMS, *Mn* ~5,000, *Xn* ~67) (Aldrich) onto the aminosilane-grafted surface to cover the whole surface, then heated at 80 °C for 4 h. Unreacted PDMS was removed by immersing the treated glass substrate in 2-propanol (Aldrich) for 5 min with sonication.

The bulk PDMS surface was prepared by spin-coating a mixture of PDMS prepolymer and curing agent (Sylgard 184, Dow Corning Chemicals) on the rectangular glass substrates. The PDMS prepolymer was mixed with the curing agent at a ratio of 10:1, and the speed of spin-coating was 2,000 rpm.

The multilayer PDMS nanobrush was prepared through partial oxidation. The monolayer PDMS nanobrush plates were exposed to UV-ozone for 60 s, which induced partial photooxidation only at the topmost region of the PDMS nanobrush monolayer, while the remaining monolayer retained the pristine PDMS nanobrush state. Partial photooxidation induced the formation of hydroxyl groups (-OH). Subsequently, the whole process was repeated to obtain the desired number of layers. Each step during this process was validated by measuring the layer thickness and water contact angle.

### Materials Characterization

The PDMS nanobrush layer thickness was measured by spectroscopic ellipsometry (SE MG-Vis 1000, Nanoview). The static/dynamic water contact angles for each PDMS surface (nanobrush and bulk) were measured by Phoenix, SEO. The chemical composition of the PDMS nanobrush layer was determined by performing X-ray photoelectron spectroscopy (XPS, K-alpha, Thermo, U.K) using Al Kα radiation (1,486.6 eV) and attenuated total reflectance Fourier transform infrared spectroscopy (ATR-FTIR, Vertex 70, Bruker). The PDMS nanobrush surface morphology was investigated by field-emission scanning electron microscopy (FE-SEM, JSM-7001F, JEOL) and atomic force microscopy (AFM, MFP-3D-SA, Asylum Research).

### Cell Culture on PDMS Surface

Brain cancer cells (SF295) were cultured with slight modifications to the standard protocol. SF295 cells were cultured in RPMI1640 growth medium containing 10% fetal bovine serum (FBS) and 1% streptomycin-penicillin (Invitrogen, CA) at 37 °C in humidified incubator of 5% CO_2_. Culture media of NIH3T3 cells was DMEM supplemented with the same amounts of FBS and antibiotics. Human mesenchymal stem cells (hMSC) and its culture media were purchased (Lonza, CA) and grown in an undifferentiated condition, following the given protocol. Cath.a-differentiated (CAD) cells were cultured in DMEM including 5% FBS and 1% streptomycin-penicillin. Its differentiation condition for neurite outgrowth was maintained in a serum-free DMEM. PDMS-coated glass substrates in 12-well plates were sterilized in 70% ethanol, washed three times with phosphate-buffered saline (PBS), and pre-incubated in a growth medium for 30 min. Then, SF295 cells were seeded at a density of 5 × 10^4^ cells per well, coated with PDMS nanobrush or bulk PDMS. To analyze cell morphologies, SF295 cells on each PDMS surface were allowed to grow for 24 h then fixed with 4% paraformaldehyde. Cells were imaged using a Nikon C1 confocal microscope (Nikon, Tokyo, Japan), Zeiss LSM 880 Microscopes (Carl Zeiss, Oberkochen, Germany) and Eclipse Ti-U microscope (Nikon). To quantify cell population on each PDMS surface, cells were allowed to grow for 1–2 days then the MTS assay was performed according to the manufacturer’s instructions (Promega, CA).

### Immunofluorescent Staining of Focal Adhesion Complex

To analyze focal adhesion complex formation, SF295 cells were plated on glass substrates treated with the PDMS nanobrush layers or bulk PDMS in 12-well plates, and allowed to grow for 24 h. Then, cells were fixed for 15 min with 4% paraformaldehyde, rinsed with PBS, and incubated with 0.1% Triton X-100 in PBS for 3–5 min. For immunofluorescent staining, fixed cells were incubated with blocking buffer (1% horse serum, 0.1% Triton X-100 in PBS) for 30 min at 4 °C. Cells were incubated with the primary antibodies, anti-Paxillin (Sigma, St. Louis, MO) and anti-FAK (Cell Signaling, Beverly, MA), anti-Vinculin (Sigma, St. Louis, MO), anti-phospho Paxillin (BD Biosciences, Franklin Lakes, New Jersey) anti-phospho FAK (BD Biosciences, Franklin Lakes, New Jersey) and with blocking buffer overnight at 4 °C. The cells were incubated with FITC-conjugated secondary antibodies (Jackson ImmunoResearch, West Grove, PA) and DAPI (Sigma) for 2 h at room temperature. All fluorescent images were acquired by a Nikon C1 confocal microscope (Nikon Instruments, Melville, NY) and processed with NIS-Elements AR 3.2 software.

Both the aspect ratio of FA complexes (cell adhesion components) and its orientation were manually measured and quantified with fluorescence images of FAK and Paxillin using ImageJ (NIH) software. ImageJ and additional plugins (CLAHE and Log3D) were also used for the analysis of both numbers and area of cell adhesion components per cell (by processing fluorescence images of FAK, phospho-FAK, Paxillin, phospho-Paxillin and Vinculin) as previously described^28^.

### Statistical Analysis

For material characterizations such as ellipsometry, IR, XPS, contact angle measurement, AFM, and SEM, measurements were replicated on at least three different samples (n_min_ = 3) and error bars denote the standard deviations (SD) of the means.

For cellular studies such as MTS assay and FA analysis, graphs display the means from triplicate independent experiments (n = 3) and error bars represent the standard errors of the mean (SEM). Statistical significance was evaluated using student’s t-test for the comparison between two groups and one-way ANOVA with Turkey’s test for multiple comparison tests. *p < 0.05, **p < 0.01, or ***p < 0.001 were considered statistically significant. Sigma stat 3.5 (SYSTAT software, USA) and Origin (OriginLab, USA) were used for quantitative data analysis.

## Supplementary information

Supplementary Information
