# Supplementary material for: Multilayer fabrication of unobtrusive poly(dimethylsiloxane) nanobrush for tunable cell adhesion
Source: Sci Rep. 2019 Feb 12;9:1834. doi: 10.1038/s41598-018-37893-w (PMC6372672; doi:10.1038/s41598-018-37893-w)
Supplement: Supplementary file 1 — Supplementary Information [file 41598_2018_37893_MOESM1_ESM.pdf]

Supplementary Information

Multilayer fabrication of unobtrusive poly(dimethylsiloxane) nanobrush for tunable cell adhesion

Soo Sang Chae[1,†], Joo Hyun Jung[2,4,†], Won Jin Choi[5], Joung Kyu Park[5], Hong Koo Baik[1,*], Jongjin Jung[3,*] and Hyuk Wan Ko[2,*]

[1]*Department of Materials Science and Engineering, Yonsei University, Seoul 03722, Korea*

[2]*Department of Biochemistry, College of Life Science and Biotechnology, Yonsei University, Seoul 03722, Korea*

[3] *Department of Chemistry, Hannam University, Daejeon 34054, Korea*

[4] *College of Pharmacy, Dongguk University, Goyang 10326, Korea*

[5]*Advanced Materials Division, Korea Research Institute of Chemical Technology (KRICT), Daejeon 34114, Korea*

[†]*These authors contributed equally to this work.*

[*]Correspondence and requests for materials should be addressed to H.W.K (email: kohw@yonsei.ac.kr), J.J (email: jongjin9@hnu.kr), or H.K.B (email: thinfilm@yonsei.ac.kr)

Supplementary Figures

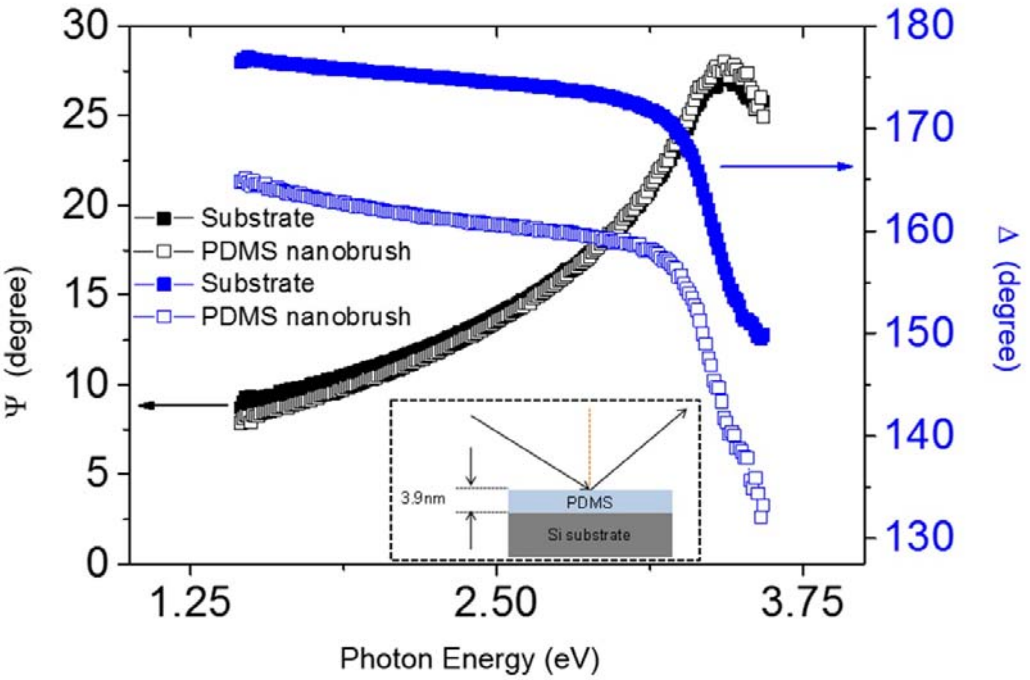

Figure S1. Thickness of the PDMS nanobrush layer. Thickness of the PDMS nanobrush layer was measured by spectroscopic ellipsometry as 3.9 nm. The measurements were replicated on at least three different samples (n_{min} = 3). The standard deviation of its measurement is depicted in the third step of Figure 1a.

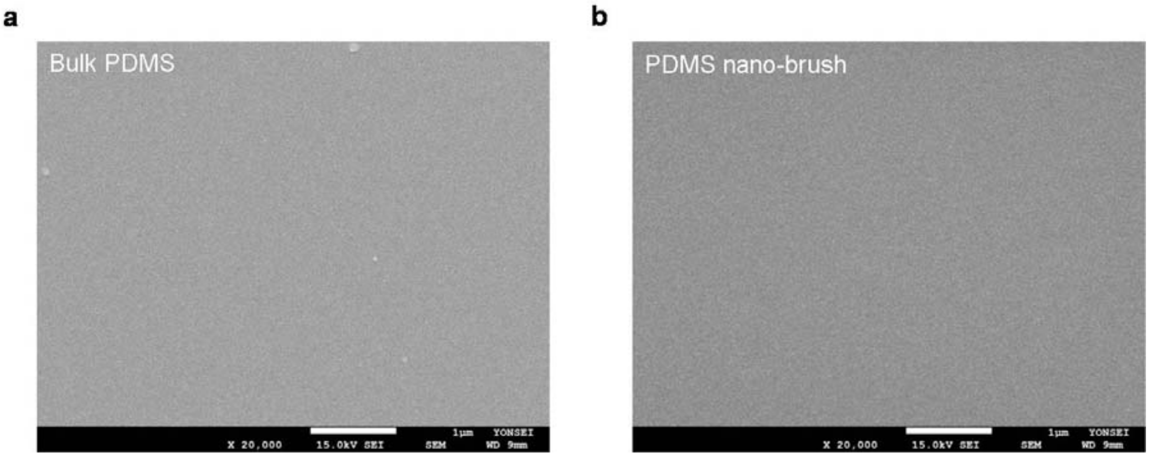

Figure S2. Scanning electron microscopy (SEM) images of (a) bulk PDMS and (b) the PDMS nanobrush layer surfaces. All data was obtained in triplicate on at least three different samples.

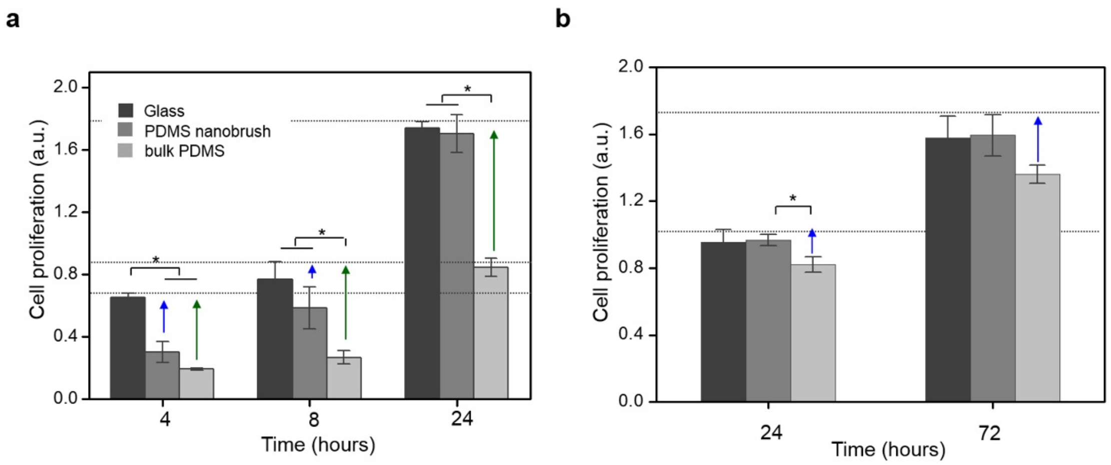

Figure S3. Cell behavior on PDMS nanobrush, glass substrate, and bulk PDMS. (a) Number of cells grown on glass, PDMS nanobrush, and bulk PDMS at indicated times (4, 8, and 24h, respectively) were assessed with the MTS assay. (b) Number of cells grown on laminin-coated glass, PDMS nanobrush, and bulk PDMS for 24 or 72h, which were assessed with the MTS assay. The graph displays the means from triplicate independent experiments; error bars represent SEM (n=3). Data were analyzed using one-way ANOVA with Tukey's test (**p*<0.05).

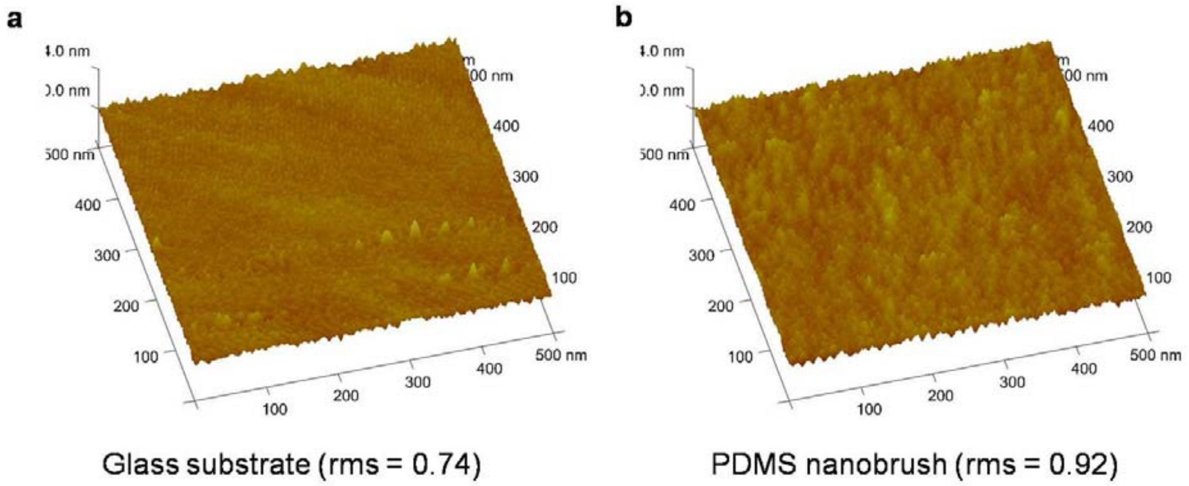

Figure S4. Atomic force microscopy (AFM) images of (a) bare glass substrate and (b) PDMS nanobrush. While the roughness corresponding to PDMS nanobrush slightly increased as a result of the sparse structure of the layer, the RMS (root mean square) of the layer could still be found within 1 nm, which might not affect to cell adhesion. All the data was obtained from three different samples.

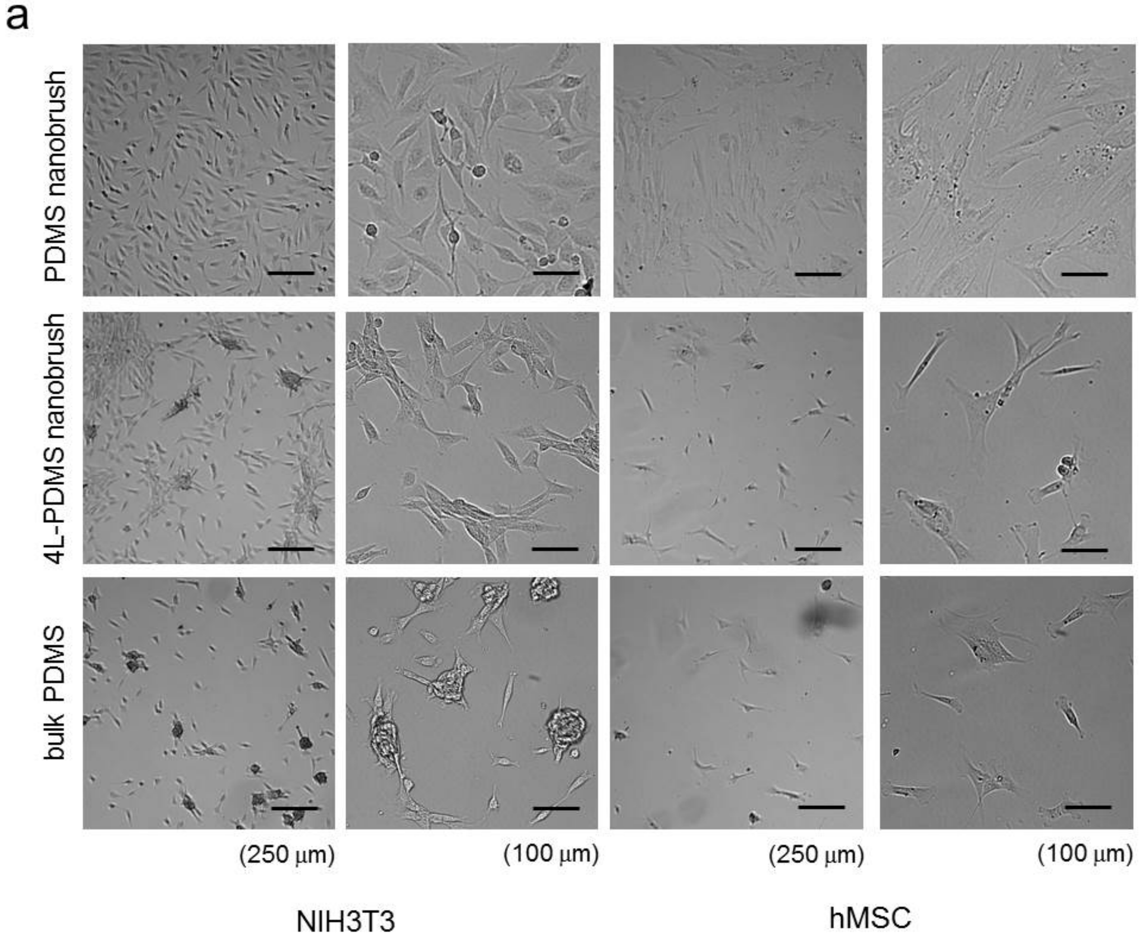

Figure S5-a. The photomicroscope images of cultured (a) NIH3T3, hMSC, and (b) CAD cells on monolayer, 4-layers PDMS nanobrush, and bulk PDMS; hMSC (human Mesenchymal Stem Cell), CAD (Cath.a-differentiated).

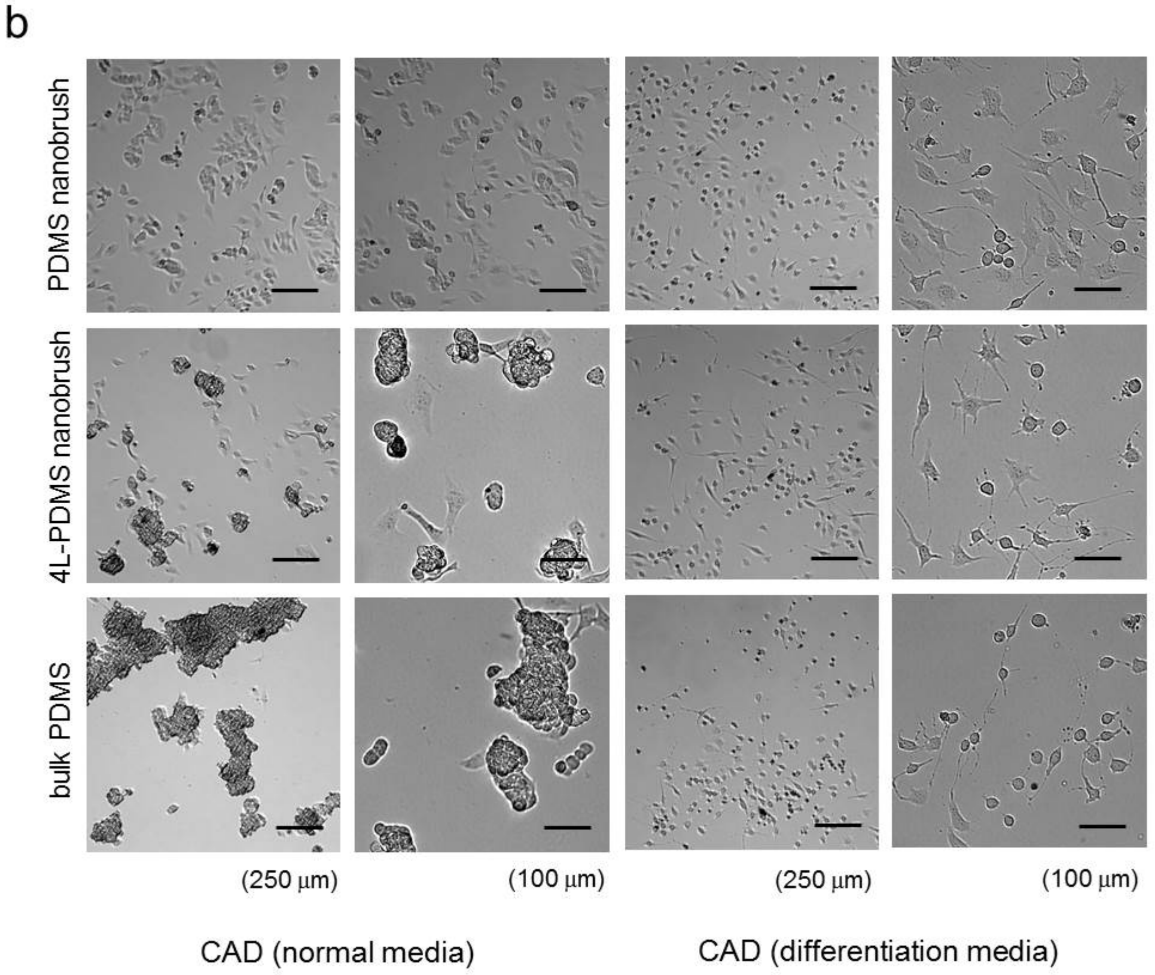

Figure S5-b. The photomicroscope images of cultured (a) NIH3T3, hMSC, and (b) CAD cells on monolayer, 4-layers PDMS nanobrush, and bulk PDMS; hMSC (human Mesenchymal Stem Cell), CAD (Cath.a-differentiated).

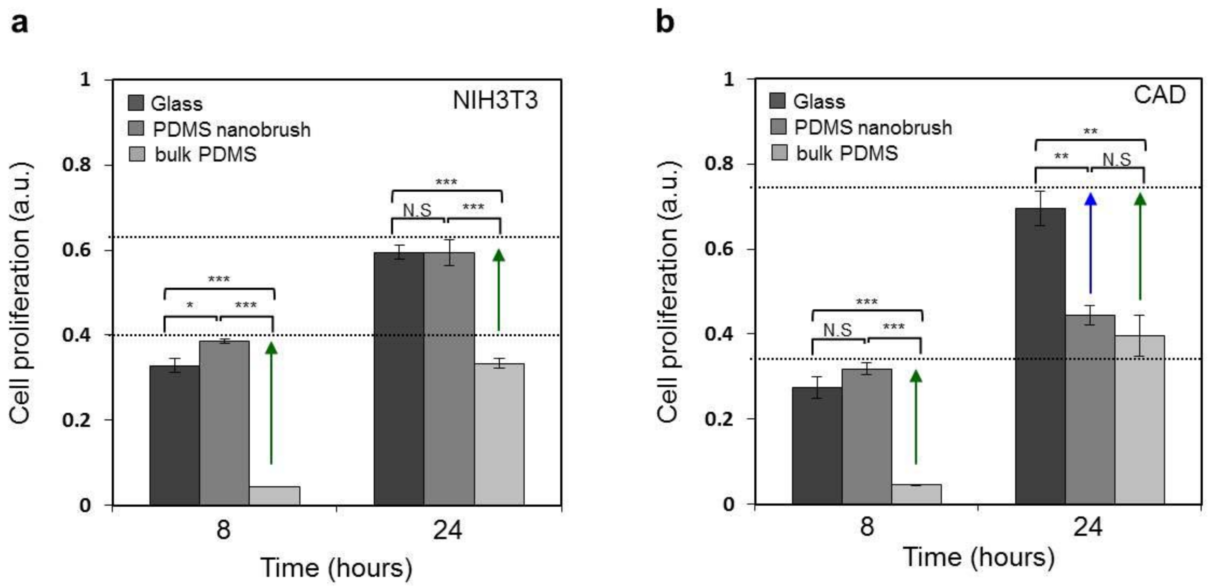

Figure S6. NIH 3T3 and CAD cell behaviors on PDMS nanobrush, glass substrate, and bulk PDMS. (a) Number of NIH 3T3 cells and (b) CAD cells grown on glass, PDMS nanobrush, and bulk PDMS in normal media at indicated times (8h and 24h, respectively) were assessed with the MTS assay. The graph displays the means from triplicate independent experiments; error bars represent SEM (n = 3). Data were analyzed using one-way ANOVA with Tukey's test (**p*<0.05, ***p*<0.01, ****p*<0.001, N.S: not significant). The quantitative result of CAD cell proliferation is quite different from other cell's results (SF295 and NIH3T3) although they show similar cell attachment, spreading and density in Figure S5.

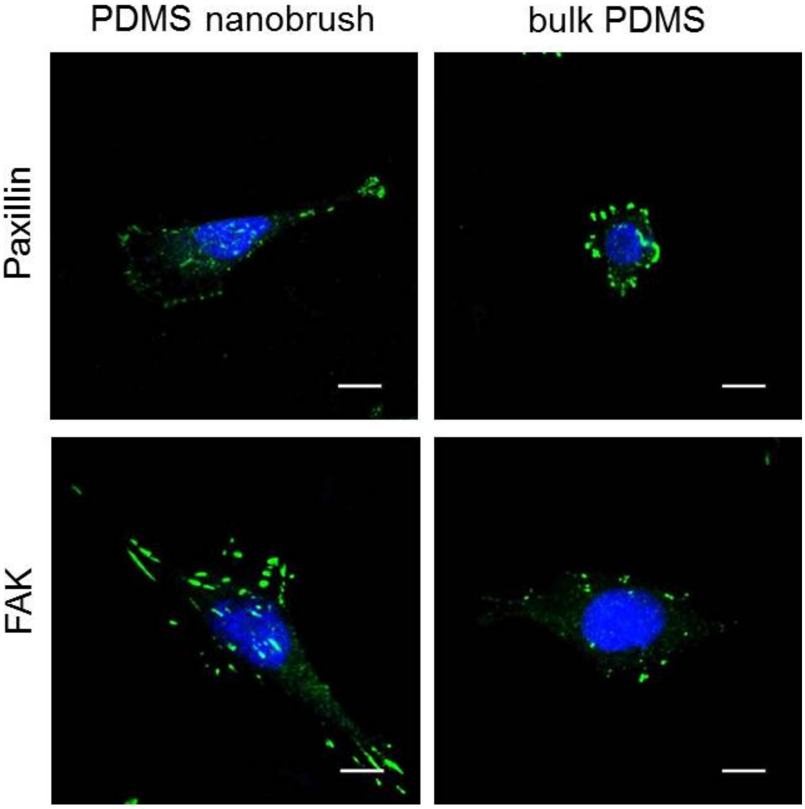

Figure S7. Magnified confocal immunofluorescence images of SF295 cells plated on the PDMS nanobrush and the bulk PDMS. Fixed cells were stained with antibodies against Paxillin (green) and FAK (green) to observe the formation of focal adhesion complexes. Nuclei were counterstained with DAPI (blue) (scale bar = 5 μm).

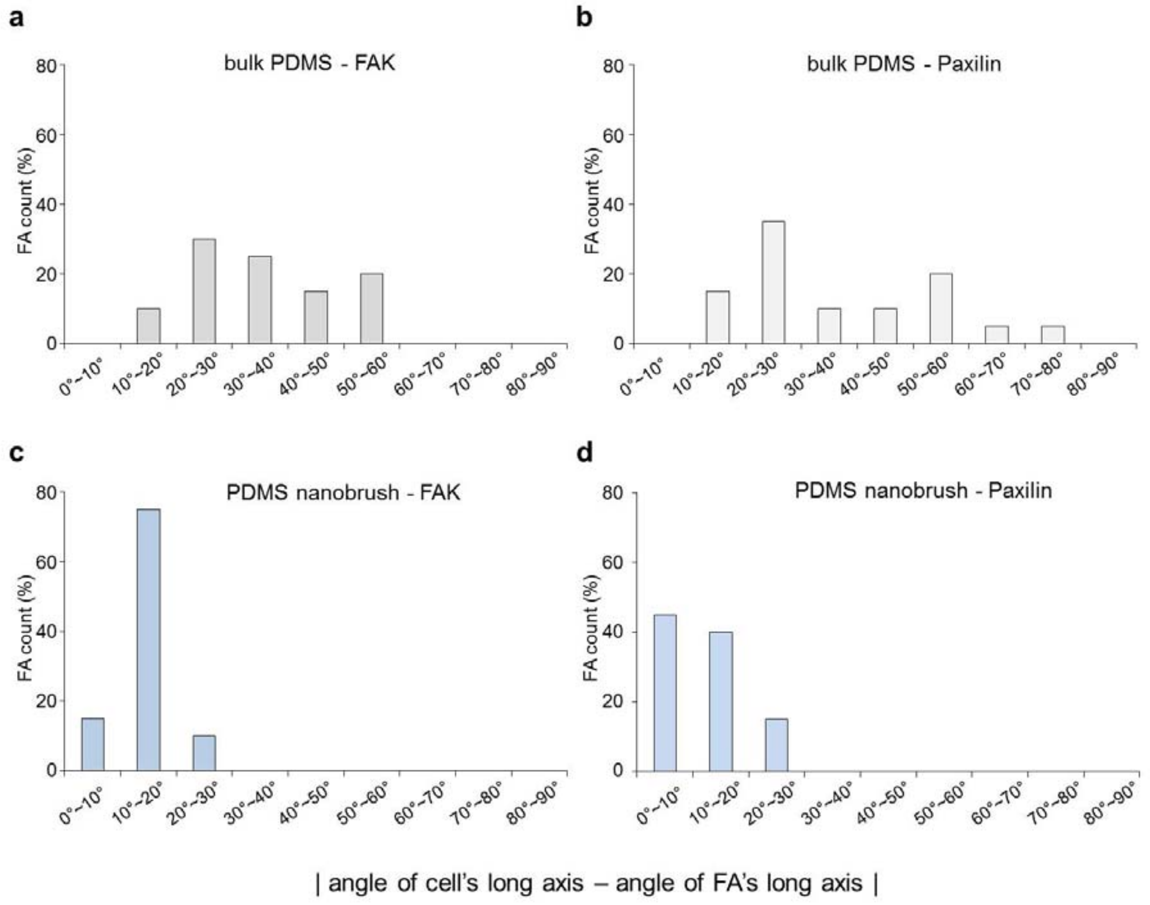

Figure S8. Quantitative analysis of relative focal adhesion (FA) complex orientation with respect to the long axis of cells grown on the PDMS nanobrush or the bulk PDMS. Quantification of FA complex angle of indicated substrates and antibodies in (a)–(d). Long axis of cells was set as 0°. Horizontal axis of histograms denotes the angle differences between cell's long axis and FA's long axis. Vertical axis shows percentages of counted FAs with different angle deviations. (a, b) Analysis of relative FA complex angle of cells grown on bulk PDMS and subsequently stained with (a) FAK or (b) Paxillin. (c, d) Analysis of relative FA complex angle for cells grown on PDMS nanobrush and subsequently stained with (c) FAK or (d) Paxillin. 20 cells were used for the analysis of FA parameters.

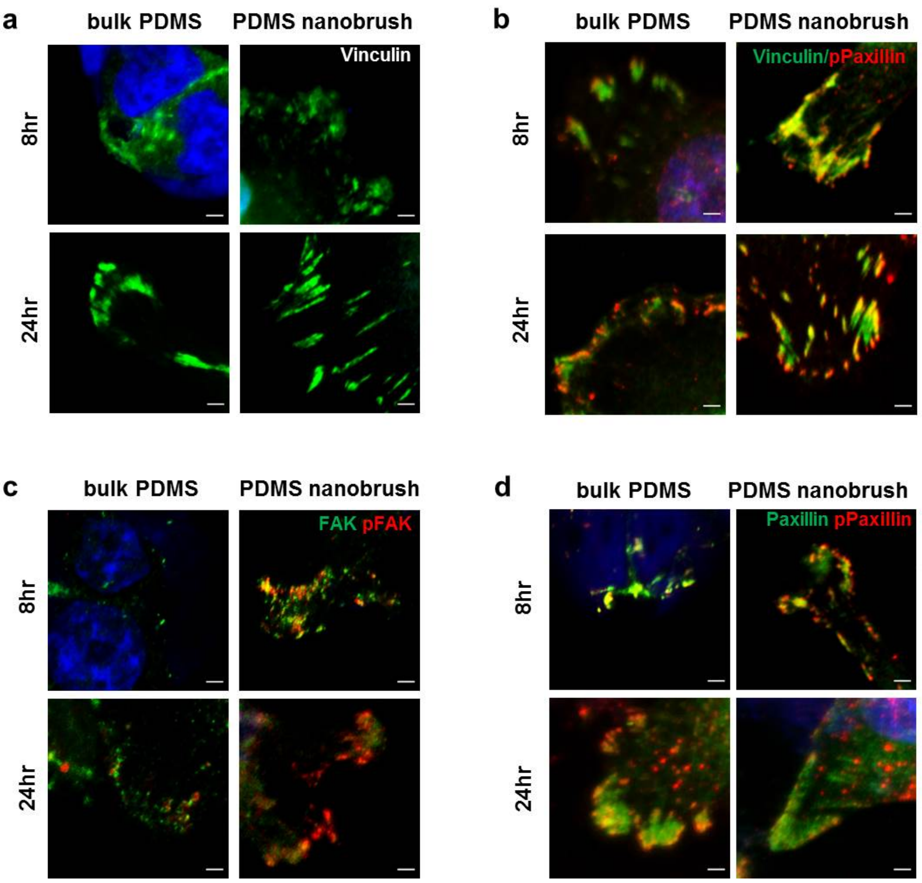

Figure S9. Localized immunofluorescence images of (a) Vinculin (green), (b) Vinculin (green) with p-Paxillin (red), (c) FAK (green) and p-FAK (red), (d) Paxillin (green) and p-Paxillin (red) on PDMS substrates at different times (8 h and 24 h) (scale bar = 2 μm).

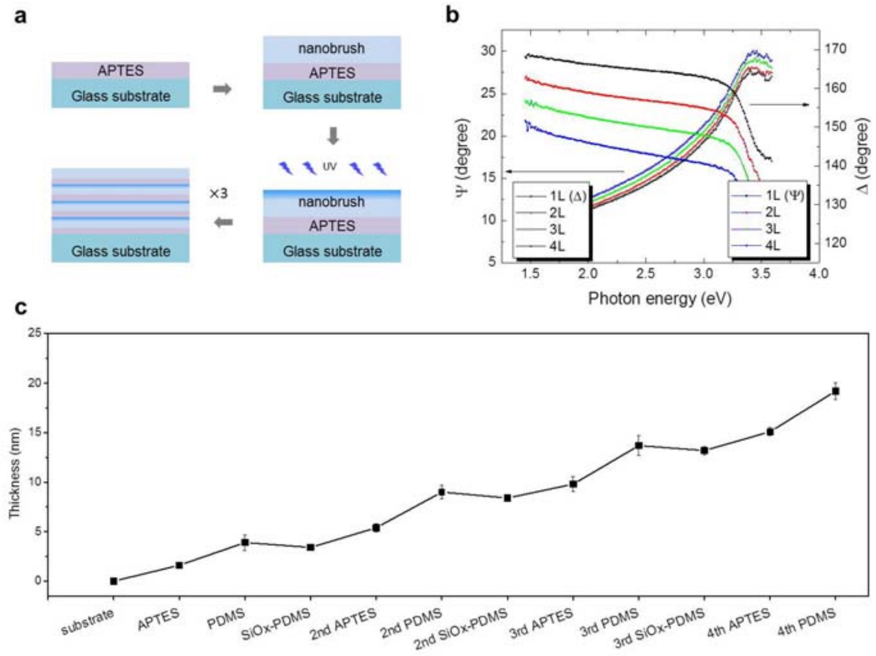

Figure S10. Fabrication of the multilayer PDMS nanobrush. (a) Fabrication schemes for multilayer deposition of PDMS nanobrush. Before deposition of a new layer, the previous PDMS nanobrush layer was partially photo-oxidized by UV exposure. (b) Spectroscopic ellipsometry measurement for multilayer deposition of PDMS nanobrush. (c) Thicknesses of the PDMS nanobrush layers are determined by fitting spectroscopic ellipsometry data with3-aminopropyltriethoxysilane (APTES) treatment and the partially oxidized state. The measurements were replicated on at least three different samples (n_{min} = 3) and the values were evaluated with standard deviation.

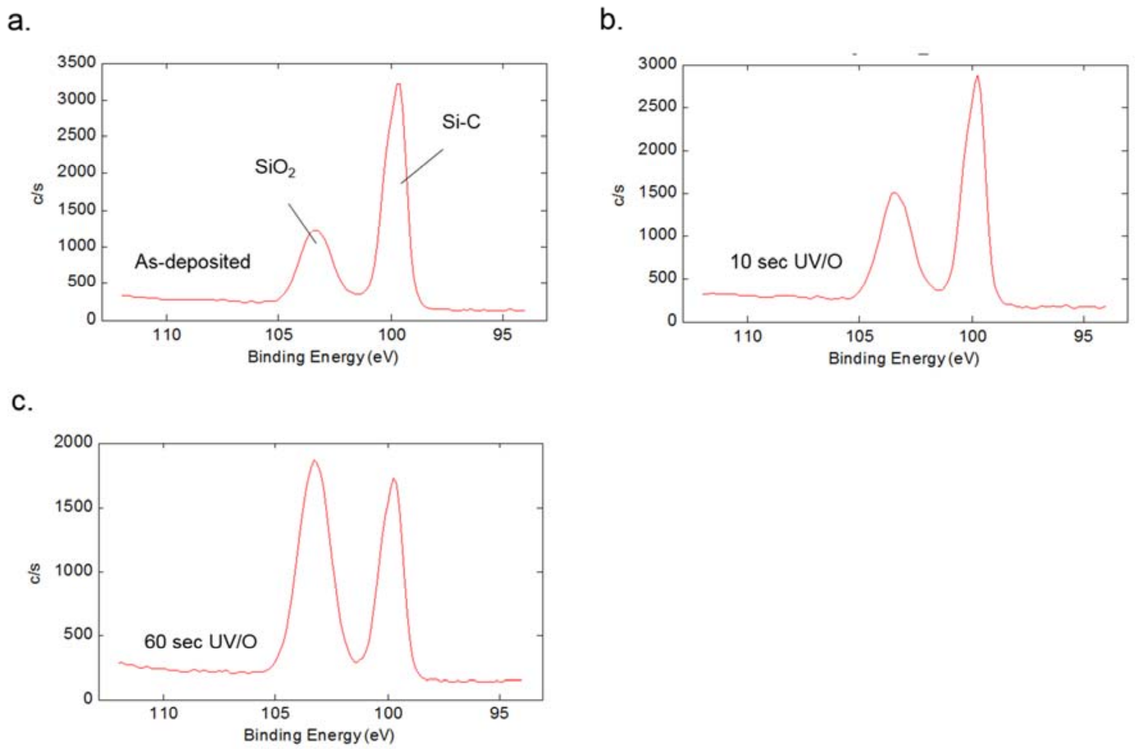

Figure S11. XPS spectra of photo-oxidized PDMS nanobrush. (a) Si 2p spectra for bare PDMS nanobrush. High intensity of Si-C peaks is originated from methyl group of bare PDMS state. (b) Increased peak intensity of SiO_2 are resulted from the formation of photo-oxidized SiO_x after10 sec exposure time of UV/O. (c) After 60 sec UV/O exposure, the intensity of SiO_2 peak is larger than that of Si-C bond, which means the formation of numerous Si-OH as an active site for 2nd layer of PDMS nanobrush. All the data were acquired from three different samples.

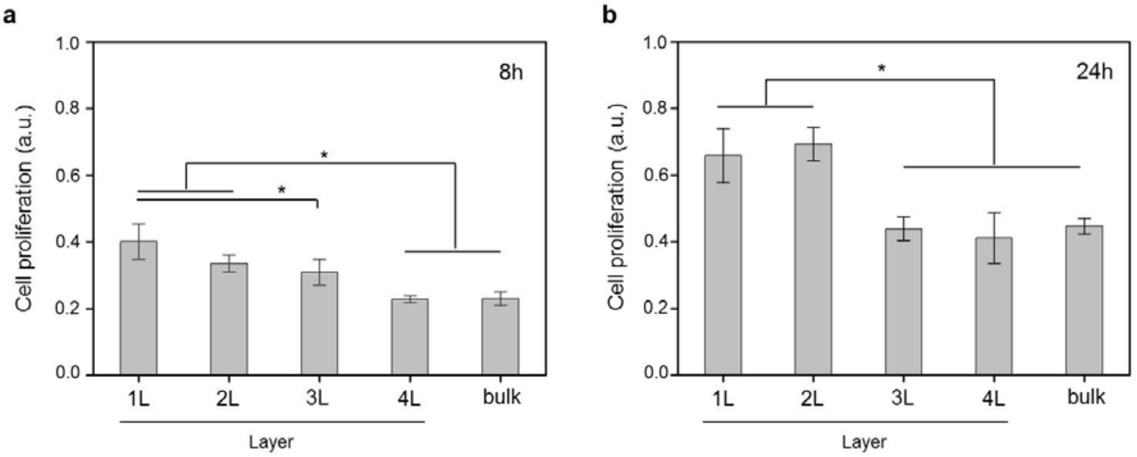

Figure S12. Number of cells grown on bulk PDMS and multilayer PDMS nanobrush substrates (monolayer ~ four layer) for both (a) 8 hours and (b) 24 hours were assessed with the MTS assay. The graph displays the means from triplicate independent experiments; error bars represent standard deviation (n = 3). Data were analyzed using one-way ANOVA with Tukey's test (*p<0.05).
